# Overcoming field variability: unsupervised domain adaptation for enhanced crop-weed recognition in diverse farmlands

**DOI:** 10.3389/fpls.2023.1234616

**Published:** 2023-08-09

**Authors:** Talha Ilyas, Jonghoon Lee, Okjae Won, Yongchae Jeong, Hyongsuk Kim

**Affiliations:** ^1^ Division of Electronics and Information Engineering, Jeonbuk National University, Jeonju-si, Republic of Korea; ^2^ Core Research Institute of Intelligent Robots, Jeonbuk National University, Jeonju-si, Republic of Korea; ^3^ Production Technology Research Division, National Institute of Crop Science, Rural Development Administration, Miryang, Republic of Korea; ^4^ Division of Electronics Engineering, Jeonbuk National University, Jeonju-si, Republic of Korea

**Keywords:** crop-weed recognition, domain adaptation, precision agriculture, artificial intelligence, crop phenotyping, agricultural operations

## Abstract

Recent developments in deep learning-based automatic weeding systems have shown promise for unmanned weed eradication. However, accurately distinguishing between crops and weeds in varying field conditions remains a challenge for these systems, as performance deteriorates when applied to new or different fields due to insignificant changes in low-level statistics and a significant gap between training and test data distributions. In this study, we propose an approach based on unsupervised domain adaptation to improve crop-weed recognition in new, unseen fields. Our system addresses this issue by learning to ignore insignificant changes in low-level statistics that cause a decline in performance when applied to new data. The proposed network includes a segmentation module that produces segmentation maps using labeled (training field) data while also minimizing entropy using unlabeled (test field) data simultaneously, and a discriminator module that maximizes the confusion between extracted features from the training and test farm samples. This module uses adversarial optimization to make the segmentation network invariant to changes in the field environment. We evaluated the proposed approach on four different unseen (test) fields and found consistent improvements in performance. These results suggest that the proposed approach can effectively handle changes in new field environments during real field inference.

## 1 Introduction

Deep Learning (DL) techniques have been successful in detecting and recognizing objects in images and videos. These techniques are now being applied to agriculture, particularly in the automatic detection and classification of weeds (Khan et al., 2020). This is a difficult problem because weeds and crops often have similar colors (green vs green), shapes, and textures (Adhikari et al., 2019; Sarvini et al., 2019). Weeds are plants that negatively impact crop growth and yields by competing for resources such as water, sunlight, air, and nutrients. They can also interfere with crop growth through the release of chemicals (Patel and Kumbhar, 2016; Iqbal et al., 2019). Effective weed control is therefore necessary to support crop growth. In addition, what is considered a weed in one setting may be a crop in another. The increasing global population, expected to reach 9 billion by 2050, will require a 70% increase in agricultural production (Radoglou-Grammatikis et al., 2020). However, the agricultural industry will face challenges such as limited cultivation land and the need for more intensive production. Climate change and water scarcity will also impact productivity. Precision agriculture can help address these challenges (Lal, 1991; Seelan et al., 2003).

Farmers must use various strategies to control weeds, including preventative measures (manual weeding), cultural techniques like field hygiene (low weed seed bank), mechanical methods like mowing and tilling, biological methods like using natural enemies of weeds (insects or grazing animals), and chemical methods such as herbicide application (Tu et al., 2001; Melander et al., 2005). Automated weed control systems, which can reduce labor costs and minimize herbicide use, have become desirable as labor costs have increased and concerns about health and the environment have grown (Durmuş et al., 2015; Nicolopoulou-Stamati et al., 2016). Moreover, due to a lack of interest among younger people in joining the agriculture industry, there is a shortage of labor (Sarvini et al., 2019). This shortage, combined with the need for efficient and cost-effective weed control, has made automated weeding methods more necessary than ever before (Lameski et al., 2018).

On other hand automated weed detection systems follow a series of steps to identify and classify weeds in images. These steps include acquiring images, pre-processing them, extracting features, and detecting and classifying weeds (Pantazi et al., 2016; Parra et al., 2020). Deep learning approaches have been successful in achieving accurate results in recognizing crops and weeds in real-world conditions (Li and Tang, 2018). The key challenge in these systems is distinguishing between crops and weeds (Khan et al., 2020; Matloob et al., 2020). These systems typically use fully convolutional networks (FCNs) to perform semantic segmentation, which involves labeling each pixel in an image with a specific class (such as crop or weed) (Parra et al., 2020; Coleman et al., 2022).

One of the main challenges in developing an automatic weed management system is accurately detecting and recognizing weeds in crops. This can be difficult because weeds and crops often have similar colors, textures, and shapes, and may appear differently at different growth stages (Sarvini et al., 2019; Khan et al., 2020; Ilyas et al., 2022). Other challenges include occlusion, variations in color and texture due to lighting and illumination, and the presence of motion blur and noise in images (Sa et al., 2016). The species of weeds can also vary based on geographical location, crop variety, weather conditions, and soil type (Kriticos et al., 2006). All of these factors can make it difficult to classify plants accurately.

Several studies have shown advancements in this area. For example, Tavakoli et al. (2021) utilized marginal loss function in CNN training for better classification. Raja et al. (2019) developed crop signaling for improved detection, and Moazzam et al. (2022) used a CNN ensemble for high accuracy detection in sesame fields. Gao et al. (2020) explored DL-based object detectors for weed detection in sugar beet fields, while Picon et al. (2022) and Peng et al. (2022) investigated synthetic images and RetinaNet adaptations, respectively, for better crop-weed recognition. However, there remains a challenge with these DL models: they often produce confident predictions on the dataset from the source domain (original farm) but underperform on data from different domains (other farms) due to domain shift (Vu et al., 2019). This is further complicated by the high cost of acquiring labeled data for each new domain, especially for semantic segmentation where each pixel must be labeled (Tranheden et al., 2021).

Recent research has explored unsupervised domain adaptation (UDA) to improve the adaptability of crop-weed segmentation systems. Gogoll et al. (2020) devised a method utilizing cycle GANs to regenerate source data in the target domain style while maintaining semantic and structural object consistency. The result was a considerable enhancement in the generalization capabilities of fully convolutional networks (FCNs), resulting in around a 10% increase in the mIOU metric on two different source-target domain pairs.

Similarly, Kendler et al. (2022) tackled the issue of low-level variability in plant disease recognition training data. By dividing images into multiple patches, they increased dataset diversity and improved CNN generalizability without needing environmental modification. This resulted in a 20% improvement in classification accuracy over the baseline. For corn yield prediction across different regions, Ma et al. (2021) presented a CNN training strategy based on unsupervised adaptive domain adversarial training. Li et al. (2021) proposed an intermediate domain approach to decrease the domain gap in maize residue segmentation. However, the application of this approach may be limited as the intermediate domain is problem-specific.

Our approach is based on the idea that the classification of a plant as a crop or weed should not depend on the farm environment, soil type, the specific sensor (camera) used, or other low-level sources of variability. These sources of variability are uninformative for crop-weed recognition, but can significantly affect the predictions of CNNs.

In this paper our aim is to reduce the domain gap between the extracted features, from source and target domain, *via* adversarially optimized deep feature alignment and entropy minimization. Additionally, we introduce a novel regularization technique to improve the convergence of CNNs. In contrast to previous UDA works, we also explore the effectiveness of few-shot training strategy in the context of UDA, called few-shot supervised domain adaptation (SDA). Few-shot SDA involves fine-tuning the model on a small amount of labeled data from the target domain to improve its performance on that domain. The main advantage of few-shot SDA is that it can be used to quickly adapt a model to a new domain with minimal labeled data.

Our main contributions can be summarized as follows:

- A deep adversarial optimized framework for UDA and few-shot SDA.
- Augmentation scheduling strategy for improved regularization and convergence.
- A versatile dataset for fine-grained crop-weed recognition collected from five different fields with different setups.

## 2 Materials

### 2.1 Dataset construction

**Figure 1:**
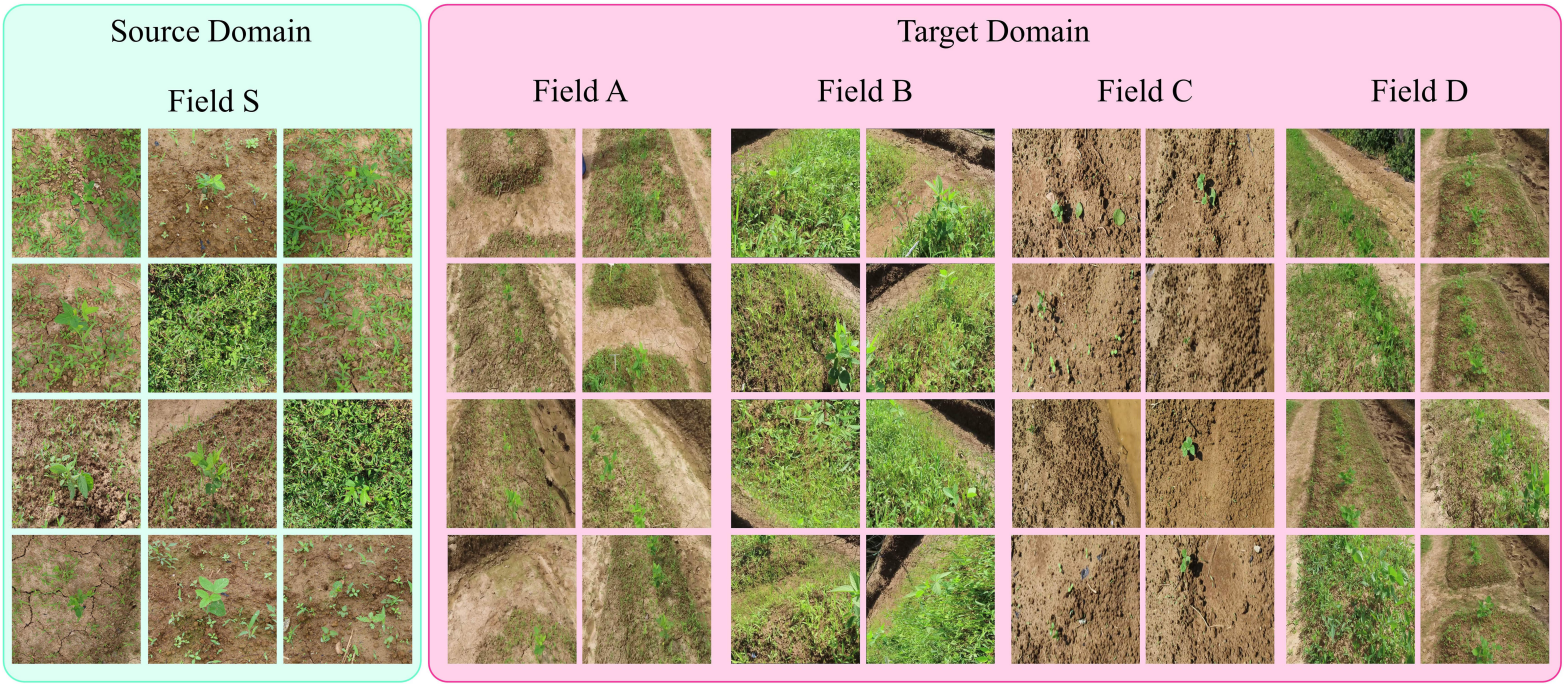
Representative images from different fields to collect data. The source domain (Field S) data is collected using handheld cameras in the form of images and are labelled by human annotators. Whereas the target domain data (Field A, B, C and D) is collected from various fields with a camera mounted on a moving platform in form of videos.

Our proposed approach was tested on a bean field dataset collected over the past one and a half years at five different locations and farms in South Korea using different image acquisition platforms. The dataset includes a number of variations in real-field conditions such as the field seeding bed (Gebrekidan, 2003), environment, weed density, plant scales, and sizes. To evaluate the performance of our approach, we selected five farms with different conditions and data variations as shown in **Figure 1** and **Table 1**.

**Table 1:** Characteristics of Crop-Weed Recognition Datasets for unsupervised domain adaptation.

Field	No. of Images	Vegetation Density	Image Acquisition Platform	Seeding System
S	1893	Varying	Stationary	Flat Bed
A	202	Medium Dense	Moving	Ridge Bed
B	79	Dense	Moving	Ridge Bed
C	199	Sparce	Moving	Flat Bed
D	157	Medium Dense	Moving	Ridge Bed

Beans are a crop that help improve soil health through nitrogen fixation, adding nitrogen back into the soil. Because of this ability, beans are often included in crop rotation plans, as nitrogen is an essential nutrient for growing strong and productive plants (Aschi et al., 2017). In countries like South Korea, where only 20% of the land is suitable for cultivation, it is especially important to use crops that can improve soil health. The collected dataset includes a bean crop and various types of weeds, but for the purposes of the crop-weed recognition task, we have grouped all the weeds into a single category. **Table 1** summarized the characteristics of the dataset.

### 2.2 Field data distribution

**Figure 2:**
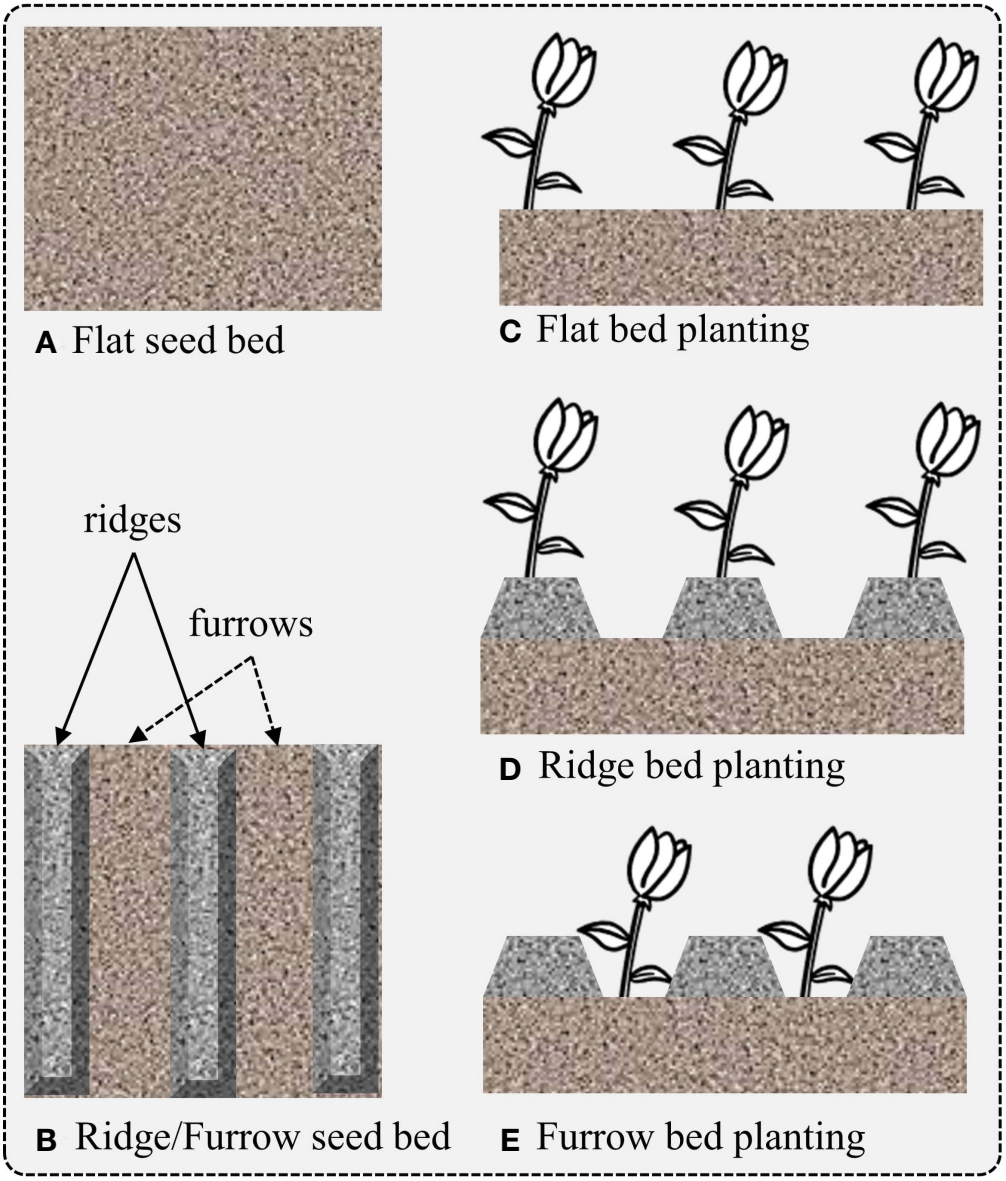
Representation of field seeding bed systems depending on the chosen planting method. Different planting methods can lead to varying crop yields for different crops. **(A)** Flat seed bed, **(B)** seeding bed with ridges and furrows, **(C)** plantation on flat seeding bed, **(D)** plantation on ridges and **(E)** plantation on furrows.

In order to make our dataset suitable for domain adaptation, such as the representation shown in **Figure 2**, we considered the case of data collected at five different locations and fields, designated as F_A_, F_B_, F_C_, F_D_, and F_S_, as shown in **Figure 1**. This is a specific example of domain adaptation across various scenarios, in which we aim to build a more robust system by transferring the visual characteristics from one field to another. In this case, we assume that the conditions of each field are different, meaning that each field may have a different weed density, seeding system, image acquisition system, and crop size. The visual characteristics of the fields used for data collection are displayed in **Figures 1**, **2** illustrates the visual attributes of various seeding bed systems across different fields.

### 2.3 Source and target datasets

In order to create the source and target datasets for unsupervised domain adaptation (UDA) in our experiments, we designated the field with the largest number of data available i.e., F_S_ as the source field, and all the other fields (F_A_, F_B_, F_C_, F_D_) as the target fields. Based on this grouping, we consider the following combinations across the five fields for evaluation: S→S, S→A, S→B, S→C, S→D. We train the network using data from the source domain (F_S_) and test it against all the target domain datasets (F_A_, F_B_, F_C_, F_D_).

## 3 Methodology

Here, we present our methodology for deep feature adaptation in context of UDA for crop-weed segmentation in unconstrained real-field environments. We also compare UDA approach with few-shot SDA for completeness. This section consists of the following sub-sections: (i) clearly defining the problem statement, (ii) introducing the architecture of the full framework, (iii) explaining the augmentation scheduling strategy which improves the performance of our framework, (iv) defining the learning objectives (loss functions), and (v) providing implementation details.

### 3.1 Problem definition

**Figure 3:**
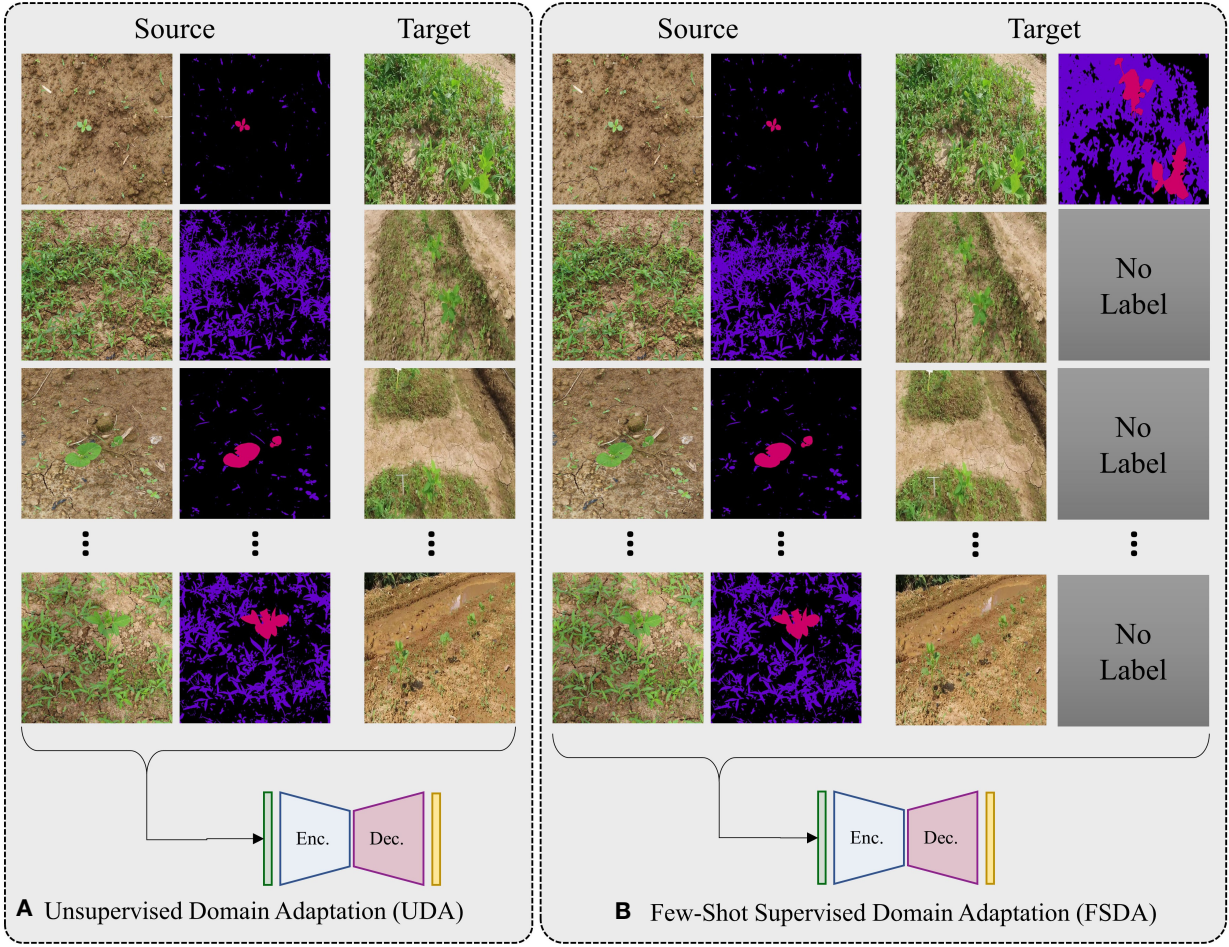
A graphical representation illustrating differences between UDA and few-shot SDA. In UDA **(A)**, there a relatively large number of unlabeled target domain data is available for use during training. In few-shot SDA **(B)**, only a small number of labeled samples (typically one or two) are available for training. The figure shows an example of 1-shot SDA as only one labelled sample is provided.

For better generalization we cast our problem as few-shot SDA because UDA can be simply defined as zero-shot SDA. Under these setting consider we are given a labelled soured dataset $D_{s}={\left\{\left(x_{i}^{s},y_{i}^{s}\right)\right\}}_{i=1}^{N}$ , where $D_{s}\subseteq \left\{F_{s}\right\}$ and N is the total number of images in *D_S_*. Similarly, we have target domain datasets, $D_{t}={\left\{\left(x_{i}^{t},y_{i}^{t}\right)\right\}}_{i=1}^{M_{t}}$ , from which we can only access *j* labelled images, here j ϵ {0,1,2,…,*M_t_*} and *M_t_* being the total number of images in *t-th* target domain dataset, and $D_{s}\subset \left\{F_{A},\;F_{B},F_{C},F_{D}\right\}$ . Here $x_{i}\in {\mathbb{R}}^{H\times W\times 3}$ is RGB-image and $y_{i}\in {\mathbb{R}}^{H\times W}$ is its corresponding label. We define *j*-shot SDA as randomly selecting *j* labelled images from each target domain datasets and using them for finetuning the network. The case of 0-shot SDA (j=0) is equivalent to unsupervised domain adaptation (UDA). For experiments we only consider *j* = 0,1,3,*M_t_*. A graphical illustration that demonstrates the distinctions between UDA and few-shot SDA is displayed in **Figure 3**.

### 3.2 Augmentation scheduling

In conventional data augmentation strategies employed for training deep neural networks, a constant probability is applied for data augmentation, which often comprises a mixture of geometric and noise transformations. However, our proposed method diverges from this practice by progressively increasing the frequency of data augmentation as training advances, with each type of augmentation treated distinctly. The concept of increasing the augmentation probability finds parallels in the training of PA-GANs (Zhang and Khoreva, 2019), where both the generator and discriminator of a GAN grow progressively. Starting at low resolution, layers are incrementally added to enhance the resolution over time, thereby enabling the model to initially learn coarse-level structures, and then gradually learn fine-level details as training continues.

In contrast, the proposed technique involves adjusting the intensity or probability of data augmentation over time, but does not involve changing the architecture of the model itself over the course of training. In the augmentation scheduling of GANs, the emphasis is on enhancing the stability and efficacy of training through gradual growth of the model’s structure. Conversely, augmentation scheduling focuses on presenting the model with an increasingly diverse and challenging array of training samples over time. While both techniques involve a form of progressive or scheduled change during training, they target different aspects of the training process. The augmentation scheduling technique is primarily about the model, while the augmentation scheduling technique is about the data.

Here we divide different augmentations into three categories depending on their characteristics:

- Geometric augmentations (G), augmentations which effect the entire image-label pair (x^s^,y^s^).
- Noise (distortion) augmentation (D), which only effect the original image (*x^s^*) and labels (*y^s^*) remain unchanged.
- Collage Augmentation (C) (Chiang et al., 2019), which generate a collage of multiple image-label pairs $\left(x_{i}^{s},y_{i}^{s}\right)$ in the dataset. Mathematically it can be expressed as,

(1) $$\left(x_{i}^{c},y_{i}^{c})=C({\left\{\left(x_{i}^{s},y_{i}^{s}\right)\right\}}_{i=1}^{M},w_{c},h_{c},b_{c}\right)$$

where, *C* represents the function to generate a collage image-label pair $(x_{i}^{c},y_{i}^{c})\;$ having width *w_c_* and height *h_c_*, of *M* images with *b_c_* being the border width (in pixels) between images.

**Figure 4:**
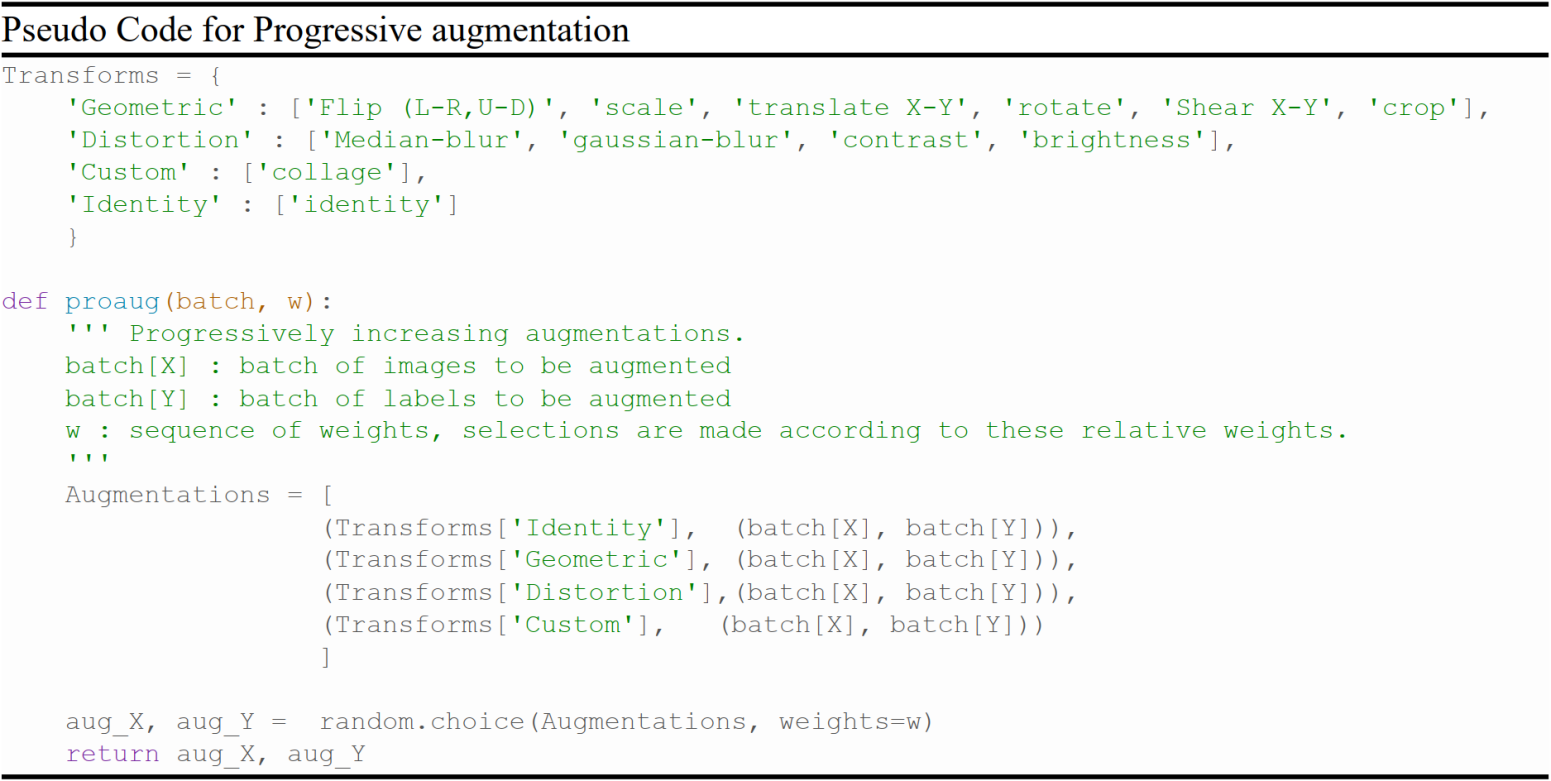
Pseudo code of Augmentation scheduling algorithm.

**Figure 5:**
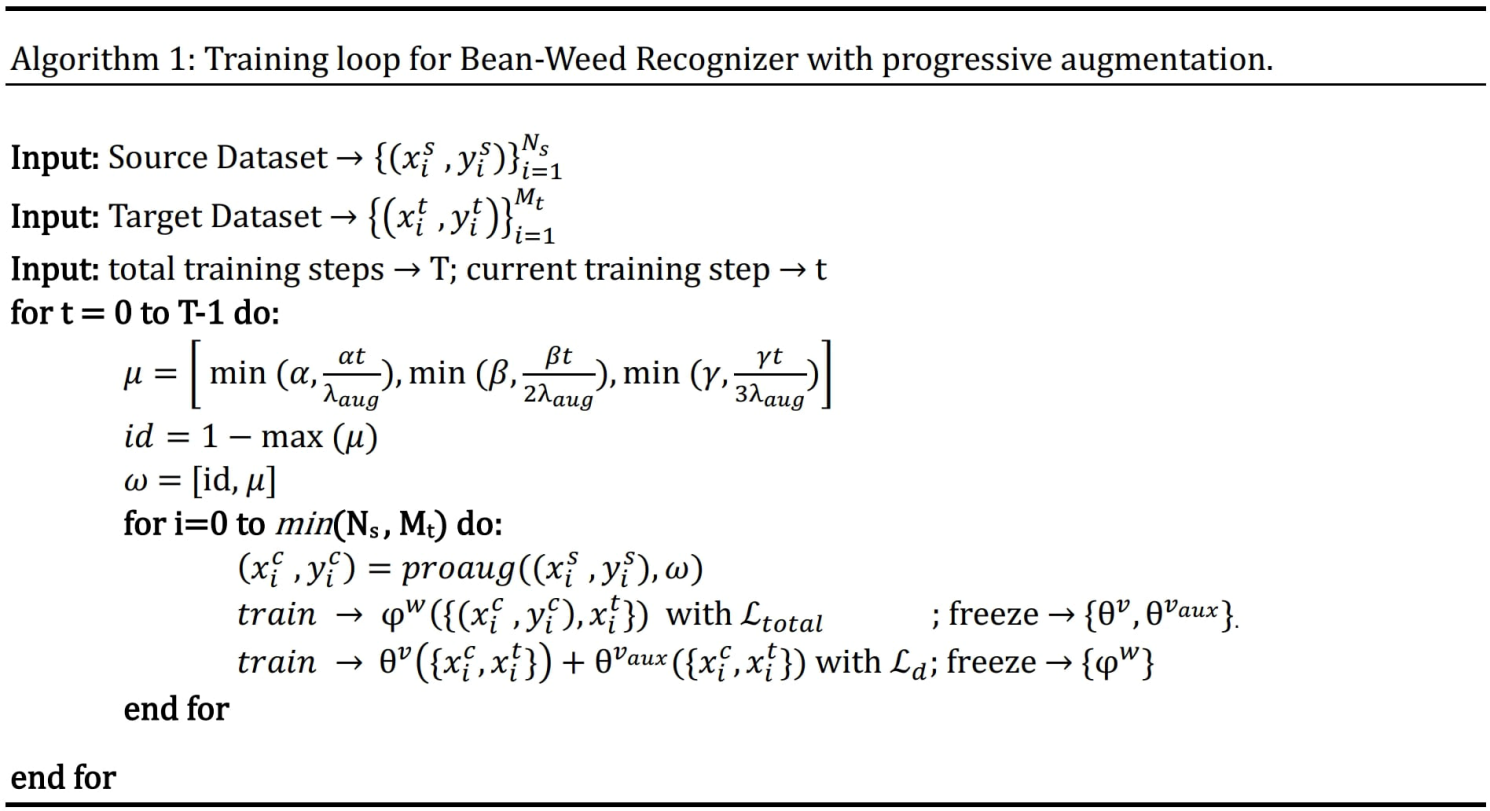
Training algorithm for proposed framework.

**Figure 6:**
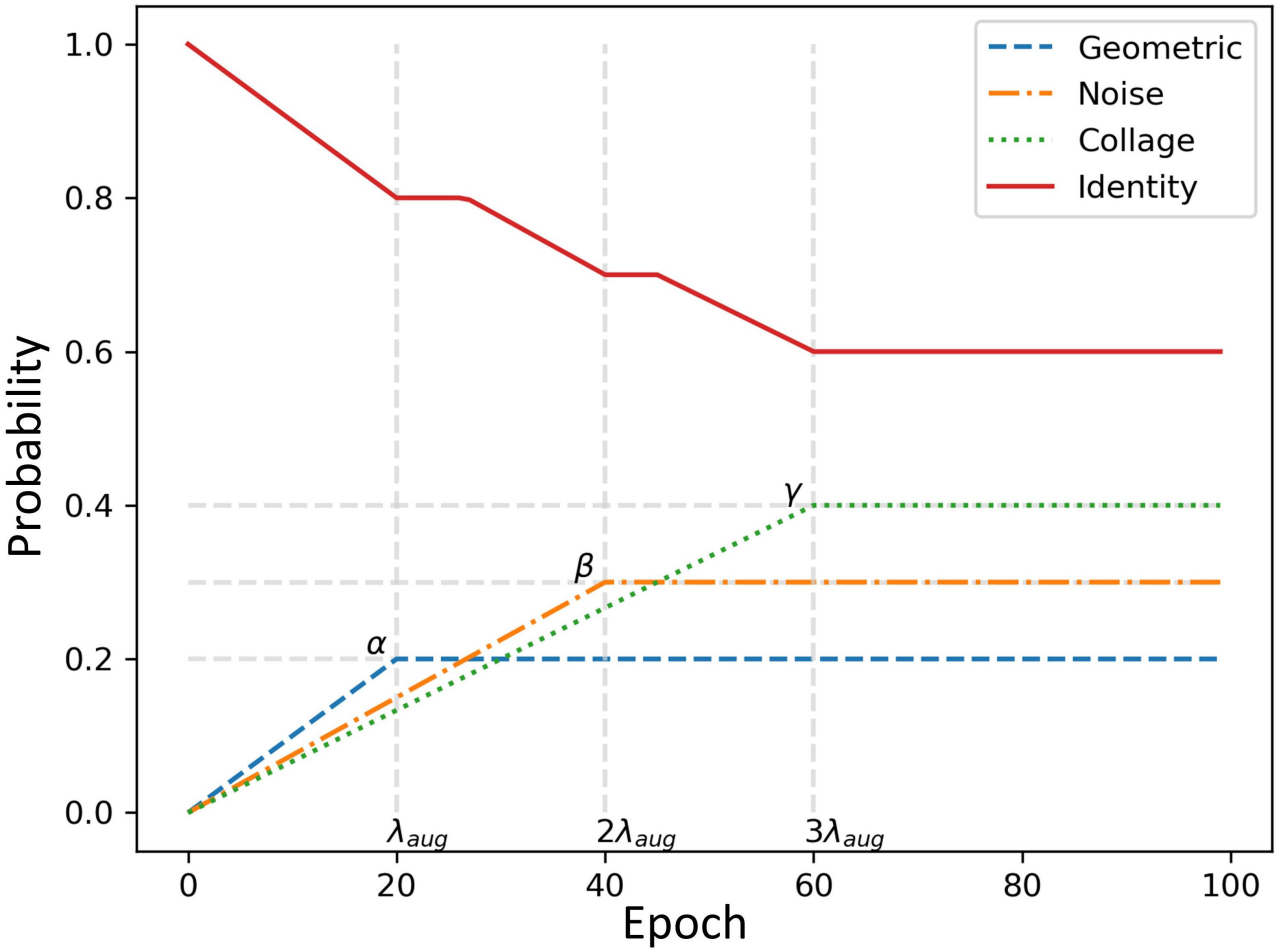
Line graph representing the changes in probabilities for each type of augmentation with training epochs for a specified set of hyperparameters, i.e., α=0.2, β=0.3, γ=0.4 and λ_aug_=20.

**Figure 7:**
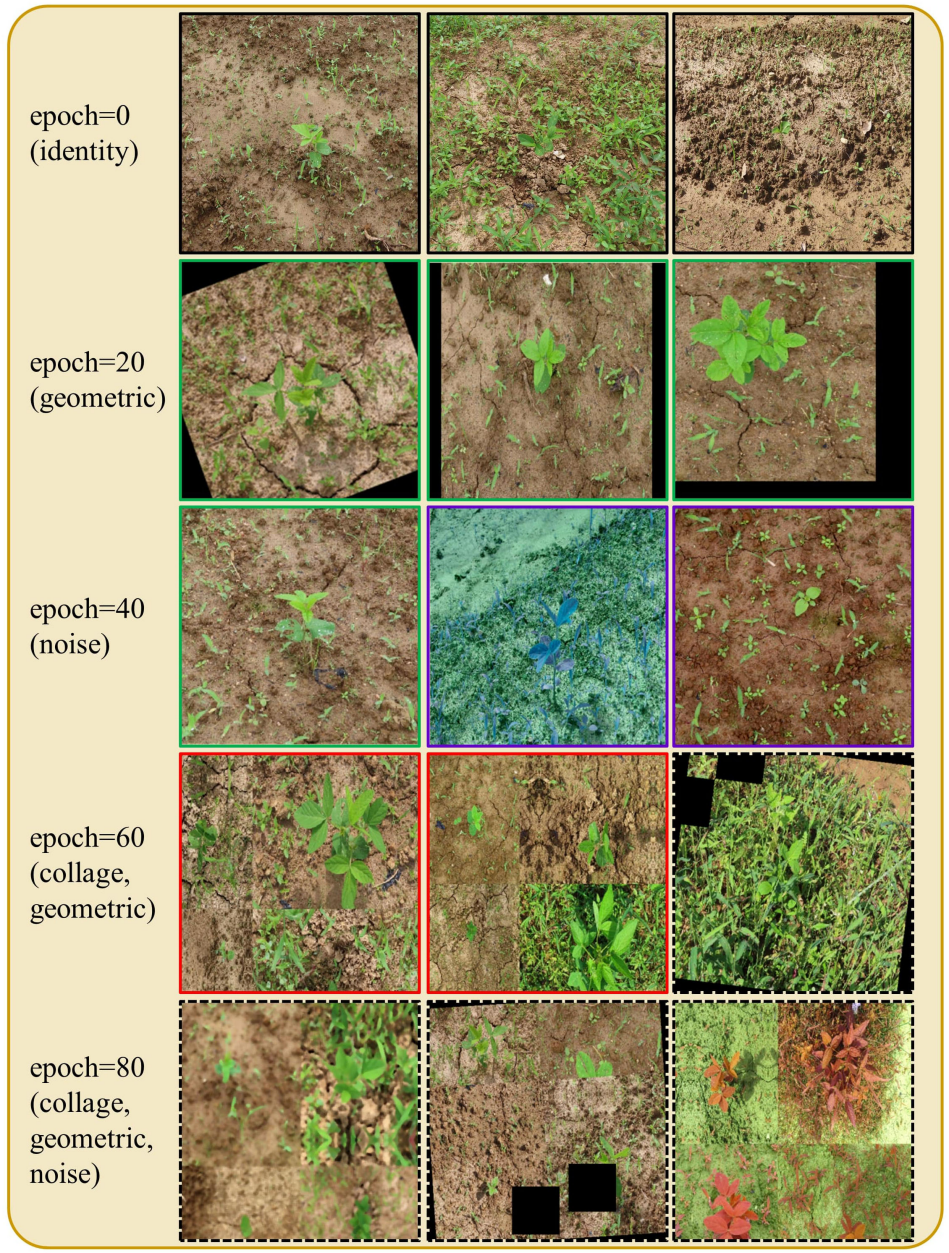
Augmentation scheduling in action: images are augmented using various combinations of augmentations at different training epochs. Black boxes depict identity augmentation, green boxes depict geometric (G) augmentation, purple boxes depict noise (D) augmentation, red boxes depict the collage (C) augmentation, and the dashed black boxes represent the application of all augmentations simultaneously.

In the early training epochs, we only use the original images (identity augmentation, i.e., id = 1) so that the network can easily and quickly learn simple representations. We only augment the source domain images. Then, we gradually increase the probability of using the other augmentations, starting with geometric augmentations and eventually using all augmentations with specified probabilities (i.e., α, β, γ > 0). These stronger regularizations make learning more difficult for the CNN and improve its robustness. The probability weights for each type of augmentation can be considered as hyperparameters (i.e., α for G, β for D, and γ for C). The pseudo code for the augmentation scheduling process is shown in **Figure 4**, and Algorithm 1 **Figure 5** summarizes the procedure for integrating augmentation scheduling into the proposed training loop of the framework. It is straightforward to adapt this to a standard training loop. Line graph in **Figure 6** shows how the probability of each type of augmentation changes with training epochs for a specified set of hyperparameters. A few examples of data samples that have been augmented using the augmentation scheduling algorithm are presented in **Figure 7**.

### 3.3 Network architecture

**Figure 8:**
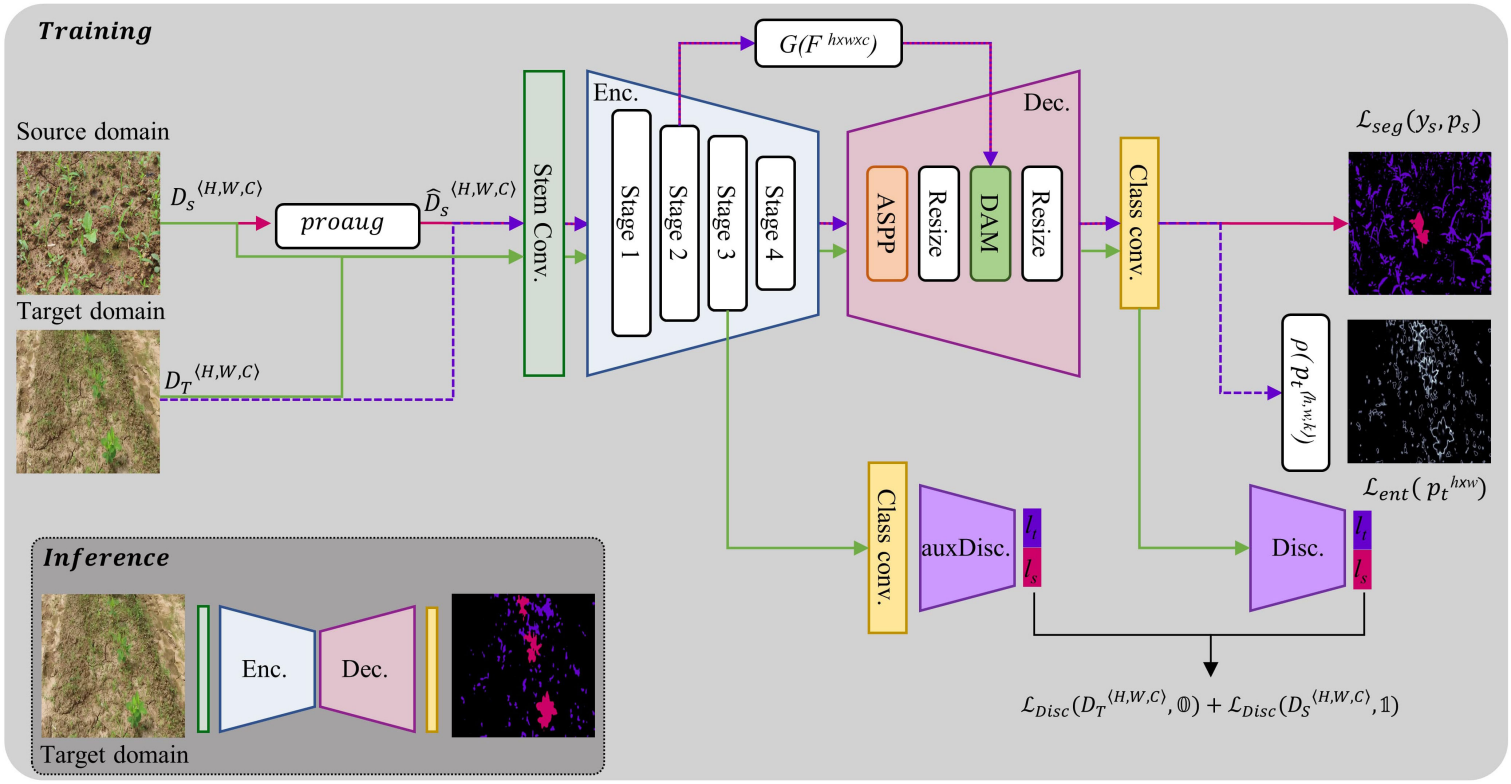
Architecture of proposed framework for UDA in crop-weed segmentation. *D_S_* and ${\hat{D}}_{s}$ represent the source domain and augmented source domain datasets respectively. During encoder-decoder training, the pink arrows depict the flow of forward and backward gradients for input from the source domain, while the purple arrows represent input from the target domain. The discriminators are kept frozen during this training step. The green arrows show the flow while the discriminators are being trained. At this stage, the encoder-decoder network is kept frozen.

The proposed framework for addressing the problem of domain shift between source and target domains is depicted in **Figure 8**. It consists of two subnetworks segmentation network and the discriminator network:

Segmentation Network - The segmentation network (φ), having learnable parameters *w*, consists of two main parts: an encoder and a decoder. The encoder is made up of a stem convolution block and four stages of feature extraction. The stem block consists of two 7x7 convolutions with a stride of 2. The subsequent four stages are composed of ConvNext blocks (Liu et al., 2022), with the number of channels in each block being N_ch_∈{192, 384, 768, 1536}, in that order. Each block is repeated N_s_ times at each stage, with N_s_∈{3, 3, 27, 3}.

The decoder also has four stages. The first stage uses an ASPP (Chen et al., 2018) module to extract multiscale features from the output of the encoder. The second stage is an upsampling module. In the third stage, the encoder’s second stage features are concatenated with the output of the second stage of the decoder through a skip connection (Ronneberger et al., 2015; Badrinarayanan et al., 2017) and are then refined by a dense attention module (DAM) (Ilyas et al., 2021). To control the flow of useful information between the encoder and decoder, the encoder’s feature maps are passed through a gating function (G), to reduce the number of feature maps and suppress low-level information, before being added *via* a skip connection. This can be represented mathematically as, $G\left(x\right)=f_{r}^{1\times 1}\left(x\right)$ , where *f* is a 1x1 convolutional filter with r channels.

Discriminator Network - PatchGAN (Isola et al., 2017) is used as a fully convolutional discriminator (θ) to classify whether incoming image features are form source domain or target domain. By evaluating smaller patches of the output features rather than the full feature map as a whole allows the PatchGAN to capture fine-grained details in the original image and make more informed decisions. Our framework uses two discriminators for deep feature alignment between the source and target domain features, with one aligning the decoder features (θ^v^, having learnable parameters *v*) and the other aligning the encoder features ( ${\text{θ}}^{v_{aux}}$ , having learnable parameters *v_aux_*). It was found to be more effective than using only one discriminator at the end of the decoder. Both discriminators (θ) consist of five layers having filter size of 4x4 and a stride of 2, with the number of channels in each layer being {64,128,256,512,1}. Each convolutional layer is followed by instance normalization and a LeakyReLU activation with a negative slope of 0.2.

### 3.4 Learning objective

Given the augmented source domain labelled pair $(x_{i}^{c},y_{i}^{c})\;$ the segmentation network (φ*^w^*) predicts a *K*-dimensional soft segmentation map $p_{i}={\text{φ}}^{w}\left(x_{i}^{c}\right)$ , where $p_{i}\in {\mathbb{R}}^{H\times W\times K}$ and K is the number of classes present in the dataset. Here each K-dimensional (pixel-wise) vector is a probability distribution over classes. The segmentation network is trained by minimizing the following cross-entropy loss between the ground truth ( $y_{i}^{c}$ ) and the predicted probability map (*p_i_*), given by equation 2.


(2) $${ℒ}_{seg}(x_{i}^{c},y_{i}^{c})=-\frac{1}{N}\sum_{i=0}^{N}\langle y_{i}^{c}.\text{log}({\text{φ}}^{w}\left(x_{i}^{c}\right))\rangle$$

For target domain samples ( $x_{i}^{t})$ as annotation ( $y_{i}^{t}$ ) are not available, hence these samples can’t be used to learn the parameters (*w*) in same way as source domain samples can be used. So, following [28] here we use entropy minimization approach to maximize prediction certainty (lowering surprise) on target domain samples. Given a target domain input ( $x_{i}^{t}$ ) we generate and entropy map (*e_i_*), where $e_{i}\in {\mathbb{R}}^{H\times W}$ shows independent pixel-wise entropies of summation of network’s predictions *p_i_* (on target domain), normalized between [0,1] range. An example of entropy map is shown in **Figure 8** and mathematically expressed by equation 3.


(3) $$e_{i}=-\frac{1}{\text{log}\left(K\right)}\sum_{k=0}^{K}{\text{φ}}^{w}\left(x_{i}^{t}\right).\text{log}\left({\text{φ}}^{w}\left(x_{i}^{t}\right)\right)$$

However, minimizing entropy directly is ineffective in low entropy regions (Yang and Soatto, 2020). So, we utilize robust entropy minimization, modified *via* carbonnier penalty function which penalizes high entropy predictions more than low entropy predictions when η > 0.5. Utilizing this modified entropy loss (*L_ent_*) we update the network’s parameters by equation 4.


(4) $${ℒ}_{ent}(x_{i}^{t})=(\frac{1}{N}\sum_{i=0}^{N}e_{i}^{2}+0.0001^{2})\eta$$

Given the class probability distributions generated from the features output by third stage of encoder and final stage of decoder, represented as $p_{i_{aux}}$ and *p_i_* respectively. These distributions are then passed on to their corresponding discriminators, denoted as ${\text{θ}}^{v_{aux}}$ and θ*^v^* respectively. The goal of these discriminators is to produce domain classification outputs, with a value of 1 indicating the source domain and 0 indicating the target domain. Both discriminators are trained using the cross-entropy loss (*L_ce_*). The overall objective for the final discriminator can be expressed as equation 5.


(5) $${ℒ}_{d}={ℒ}_{ce}\left({\text{θ}}^{v}(x_{i}^{c}),\;1\right)+{ℒ}_{ce}\left({\text{θ}}^{v}(x_{i}^{t}),\;0\right)$$

Similarly, an equation can be written for the auxiliary discriminator ( ${ℒ}_{d_{aux}}$ ), resulting in the total discriminator loss.


(6) $${ℒ}_{D}={ℒ}_{d}+\lambda_{aux}{ℒ}_{d_{aux}}$$

Now, the adversarial objective for training segmentation network can be written as,


(7) $${ℒ}_{adv}={ℒ}_{ce}\left({\text{θ}}^{v}(x_{i}^{t}),\;1\right)$$

Both the segmentation and discriminator networks are jointly trained in each iteration. During training, the supervised segmentation loss for source domain samples and unsupervised entropy loss for target domain samples are jointly optimized. The adversarial loss trains the segmentation network to deceive the discriminator by maximizing the probability of target predictions being considered as source predictions. This is achieved by minimizing the cross-entropy loss between the discriminator’s predictions for target images and the label of the source domain, which is 1. Therefore, the total loss becomes,


(8) $${ℒ}_{total}={ℒ}_{seg}+\lambda_{ent}{ℒ}_{ent}+\lambda_{adv}{ℒ}_{adv}$$

In the few-shot SDA scenario, where we have *j* labelled images from the target domain, which are used to fine-tune our model. In addition to the entropy minimization loss described in equation 4, we also incorporate a cross-entropy loss similar to equation 2 for these *j* examples. Let’s denote these labeled examples from the target domain as $D_{t}={\left\{\left(x_{i}^{t},y_{i}^{t}\right)\right\}}_{i=1}^{M_{t}}$ where *i* ranges from 1 to *j*. The additional cross-entropy loss for these samples can be expressed as:


(9) $${\hat{ℒ}}_{seg}(x_{i}^{t},y_{i}^{t})=-\frac{1}{j}\sum_{i=0}^{j}\langle y_{i}^{t}.\text{log}({\text{φ}}^{w}\left(x_{i}^{t}\right))\rangle$$

Therefore, in the case of *j*-shot SDA, the total loss would be updated to:


(10) $${ℒ}_{total}={ℒ}_{seg}+\lambda_{seg}{\hat{ℒ}}_{seg}+\lambda_{ent}{ℒ}_{ent}+\lambda_{adv}{ℒ}_{adv}$$

where ${\hat{ℒ}}_{seg}$ corresponds to the supervised segmentation loss for the *j* labeled target domain samples, and λ*_seg_* is a weight hyperparameter to balance this new term. The model is then jointly optimized for the supervised segmentation loss on both source domain and *j* labeled target domain samples, unsupervised entropy loss for the remaining unlabeled target domain samples, and adversarial loss.

In this way, we effectively use the limited labeled data available in the target domain to guide the model’s adaptation process, while still leveraging the entropy minimization approach for the unlabeled target domain data.

### 3.5 Implementation details

In our implementation we used the PyTorch toolbox and a single NVIDIA RTX-3090 GPU, which has 24GB of memory. The source dataset, which contains a large number of images, was split into a 80% train-validation set and a 20% test set. The target datasets were split into a 70% training set (used only in the case of supervised training for comparison) and a 30% test set.

For training the segmentation network, we employed the SGD optimizer with a weight decay of 5x10^-4^. For training the discriminators, we used the Adam optimizer with a momentum value of 0.9 and 0.99. We used a cosine decay policy for the segmentation network, with a learning rate of 0.001 and warm start for the first 1000 iterations. For the discriminators, we used a polynomial decay policy with an initial learning rate of 10^-4^. A detailed list of the hyperparameter settings for the augmentation scheduling and loss function weights can be found in **Table 2**.

**Table 2:** Hyperparameter settings for proposed framework.

Hyperparameter	Value	Hyperparameter	Value
α	0.3	η	2.0
β	0.3	λ*_aux_*	0.4
γ	0.3	λ*_ent_*	0.5
λ*_aug_*	20	λ*_adv_*	0.4
λ*_seg_*	2		

## 4 Results and discussion

The performance of the proposed method for crop-weed recognition in bean fields was evaluated using the same field data distribution and source and target data splits described in Section 2. To thoroughly evaluate the proposed method, we employed widely used semantic segmentation frameworks, including DeepLab-v3+ and PSPNet (Zhao et al., 2017), with ResNet-101 (He et al., 2016), Xception-71 (Chollet, 2017), and ConvNext-L backbones as baselines. The results of our proposed method were compared with these baselines under the same operating conditions.

Firstly, we compared the performance of the proposed framework with traditional segmentation models and other recent unsupervised domain adaptation (UDA) methods. The results indicated that our proposed method performed competitively with these models. Furthermore, we demonstrated how the use of augmentation scheduling further improved the performance of our network. We also conducted ablation experiments to highlight the improvement in results achieved by using augmentation scheduling in comparison to vanilla augmentation.

Lastly, we compared the results of our proposed UDA method under both few-shot self-supervised domain adaptation (SDA) and fully supervised settings. The results showed that our proposed method performed well under both settings and yielded promising results. We evaluate the effectiveness of the proposed framework as well as compare it with other networks utilizing the Intersection-over-Union (IoU) metric, defined by equation 11.


(11) $$mIoU=\frac{1}{N}\sum_{i=0}^{N}\left|\frac{y_{i}\cap^{}p_{i}}{y_{i}\cup^{}p_{i}}\right|$$

where *y_i_* and *p_i_* represent the ground-truth and predicted segments, respectively.

### 4.1 Source training only

In the first experiment, we trained semantic segmentation architectures in a simple supervised fashion on the source field (F_S_) dataset and compared their performance. In this experiment, all models were trained on the source field dataset and results are reported on its test split (S→S), as shown in **Table 3**. PSPNet showed the worst performance among all other models when using the same backbone (ResNet-101), while DeepLab-v3+ with Xception-71 backbone performed better than PSPNet. Additionally, integrating the proposed modified decoder into the best-performing model (DeepLab-v3+ with ResNet-101) further boosted performance. It is worth noting that no data augmentation was used in these experiments.

**Table 3:** A comparison of the experimental results on a crop-weed segmentation dataset between traditional semantic segmentation and UDA methods with the use of Vanilla Augmentation.

Backbone	Framework	Method	mIOU (%)					
			S→S	S→A	S→B	S→C	S→D	Average
ResNet-101	PSPNet	–	74.05	66.34	56.83	38.62	52.88	53.66
Xception-71	DeepLab V3+	–	78.7	67.63	59.19	43.05	47.68	56.13
ResNet-101	DeepLab V3+	–	79.57	68.9	59.34	40.43	54.34	57.75
ResNet-101	Proposed	–	80.02	68.53	58.88	41.35	53.7	57.36
ResNet-101	DeepLab V3+	Vu et al., (2019)	**80.95**	63.66	55.62	52.31	47.62	54.80
ResNet-101	DeepLab V3+	Tsai et al. (2018)	75.94	75.57	65.23	45.65	**66.7**	63.28
ResNet-101	DeepLab V3+	Proposed	79.55	74.1	62.6	49.56	64.48	62.18
ConvNext-L	Proposed	Proposed	79.93	**75.67**	**68.54**	**52.36**	63.34	**64.97**

Bold and underlined values represent best and second-best values, respectively.

Under the source training only (STO) setting, we also tested the segmentation performance of only source-trained models on other target domain fields (i.e., F_A_, F_B_, F_C_, F_D_). The results are reported in **Table 3** under columns S→T, where T∈{A, B, C, D}. It can be seen from the table that, even though using better segmentation architectures resulted in considerably better performance on the F_S_ dataset, the results on the target domain fields did not improve and even got worse in some cases (e.g., the mIOU of field *A* and *C* decreased when using DeepLab-v3+ (ResNet-101) and proposed decoder). This demonstrates the need for unsupervised domain adaptation (UDA) approaches in the field of precision agriculture.

### 4.2 Unsupervised domain adaptation

**Figure 9:**
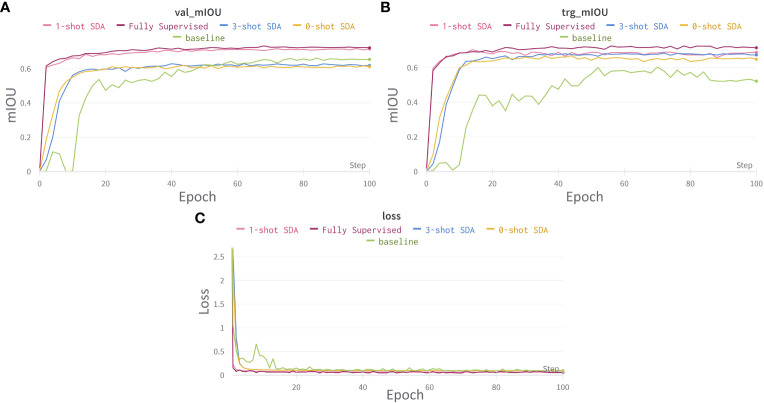
Training and loss curves for cross domain adaptation. **(A)** learning curve for mIOU score on source domains validation set. **(B)** learning curve for mIOU score averaged over all target domain test sets. **(C)** Segmentation network’s loss curves. Best viewed in color.

In our unsupervised domain adaptation experiments, we used the same data pairs as in previous experiments. We applied the augmentation scheduling algorithm with the hyperparameter values listed in **Table 2**. The results of these experiments are shown in **Tables 3**, **4**, with and without augmentation scheduling. Overall, we observed a significant improvement in the mIOU score for bean-weed recognition compared to STO methods (as seen in **Table 3**’s top four rows). Our proposed deep feature alignment method without augmentation scheduling performed better on average than previous STO and UDA methods. As shown in **Figure 9**, using proposed deep feature alignment method resulted in a noticeable improvement in performance compared to using only STO. Additionally, incorporating augmentation scheduling further increased the performance of all models. Specifically, our proposed segmentation model that uses both deep feature alignment and augmentation scheduling outperformed previous best STO models by 8% and previous best UDA methods by 7%. The performance gap was even greater on target fields FA and FD, with improvements of 5.42% and 8.1% respectively.

**Table 4:** A comparison of the experimental results on a crop-weed segmentation dataset between traditional semantic segmentation and UDA methods with the use of Augmentation scheduling.

Backbone	Framework	Method	mIOU (%)					
			S→S	S→A	S→B	S→C	S→D	Average
ResNet-101	PSPNet	–	78.31	77.07	67.36	47.39	62.33	63.53
Xception-71	DeepLab V3+	–	82.5	78.16	62.05	48.49	63.46	63.04
ResNet-101	DeepLab V3+	–	84.81	75.75	69.85	56.88	60.69	65.79
ResNet-101	DeepLab V3+	Vu et al. (2019)	83.99	80.6	73.4	60.86	71.7	71.39
ResNet-101	DeepLab V3+	Tsai et al. (2018)	79.8	78.75	70.8	50.67	79.98	70.05
ResNet-101	DeepLab V3+	Proposed	83.97	84.2	73.52	**61.71**	74.7	73.53
ConvNext-L	Proposed	Proposed	**88.5**	**91.02**	**83.11**	60.3	**81.1**	**78.88**

Bold and underlined values represent best and second-best values, respectively.

### 4.3 Few-shot supervised domin adaptation

**Figure 10:**
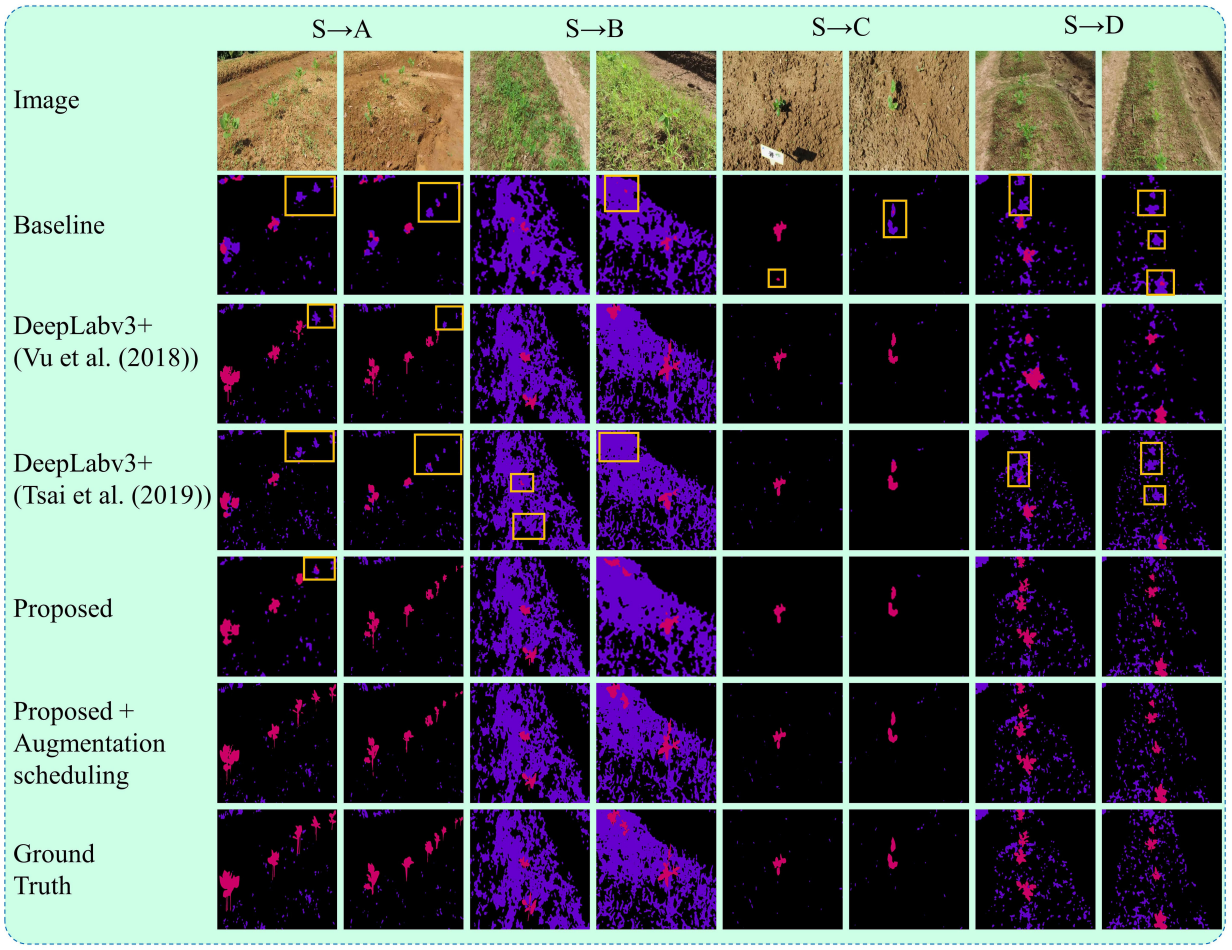
Segmentation results on datasets from the target domain under UDA setting. The results include the baseline method, DeepLabv3+ with the UDA algorithms from Vu et al., (2019) and Tsai et al. (2018), and the proposed method with and without augmentation scheduling. The ground truths are also displayed for comparison. Each target field **(A–D)** has two columns, with each column representing a different testing field with varying farm environments such as weed density, seeding bed types, plant sizes, and camera viewing angles. Boxes highlight the crops being misclassified as weeds.

In this section, we compared our approach with other conventional few-shot SDA and fully supervised methods. The results are summarized in **Table 4**. All experiments were conducted under the same conditions. For the fully supervised training, all models were trained using training splits of both the target and source dataset as described in subsection 3.5 (Implementation Details). Under these conditions, our proposed segmentation network showed an improvement of 3% in the mIOU score compared to the DeepLabv3+ model, indicating its superior feature extraction ability. For the few-shot SDA experiments, the model’s parameters were fine-tuned using a small number of labeled samples from the target domain. As shown in **Table 5**, using only one labeled sample (1-shot), our model achieved an accuracy that was almost similar to that of the fully supervised model (80.53% vs 83.6%). Additionally, our proposed method consistently outperformed other SDA methods throughout the few-shot experiments. As seen in **Table 5**, our method exceeded the best-performing few-shot SDA methods by 2.5% (0-shot), 3.0% (1-shot), and 2.2% (3-shot) for bean-weed recognition. **Figure 10** compares the visualization results, demonstrating that our method showed significant improvements in recognizing crops and weeds.

**Table 5:** Comparison of mIOU scores for few-shot SDA models with varying values of *k* against fully supervised models.

Backbone	Framework	Method	Strategy	mIOU (%)					
				S→S	S→A	S→B	S→C	S→D	Average
ResNet-101	DeepLab V3+	Vu et al. (2019)	0-shot	83.99	80.6	73.4	60.86	71.7	71.39
ResNet-101	DeepLab V3+	Tsai et al. (2018)	(UDA)	79.8	78.75	70.8	50.67	79.98	70.05
ConvNext-L	Proposed	Proposed		88.5	91.02	83.11	60.3	81.1	78.88
ResNet-101	DeepLab V3+	Vu et al. (2019)	1-shot	86.13	88.71	83.59	58.89	75.05	76.56
ResNet-101	DeepLab V3+	Tsai et al. (2018)	(SDA)	76.49	85.01	73.02	61.59	74.9	73.63
ConvNext-L	Proposed	Proposed		88.7	92.53	84.25	62.5	80.87	80.53
ResNet-101	DeepLab V3+	Vu et al. (2019)	3-shot	88.57	89.66	84.17	60.69	78.62	78.28
ResNet-101	DeepLab V3+	Tsai et al. (2018)	(SDA)	77.36	86.07	75.49	62.99	76.31	75.19
ConvNext-L	Proposed	Proposed		89.01	92.67	85.6	62.9	82.43	81.4
ResNet-101	DeepLab V3+	–	Fully	90.56	93.25	83.5	61.56	83.42	80.43
ConvNext-L	Proposed	–	supervised	**91.62**	**95.24**	**85.48**	**68.67**	**85.39**	**83.69**

Bold and best results. Underlined values represent best results in their respective settings.

### 4.4 Vanilla vs. scheduled augmentation

In these experiments, we verify the superior performance of the proposed augmentation scheduling over vanilla augmentation, and the results are summarized in **Tables 4**, **6**. For these experiments we use proposed framework under UDA (0-shot SDA) settings. We experimented with different augmentation probabilities and found that augmenting 30% of all samples during each epoch produced the best results. Starting from the baseline (no augmentations), we first performed random geometric augmentations (G) and observed performance improvement. Then, we performed noise (D) and collage (C) augmentations one by one to see further improvements. A significant increase in performance, 55.36% (baseline) to 71.28%, can be seen when using collage augmentation (C), indicating that the collage augmentation improves the network’s generalization on other domains as well. Next, we combined these augmentations at a constant probability (0.3) throughout the training process. It can be seen from **Table 2** that performing all augmentations in combination considerably improved the framework’s performance compared to the baseline.

**Table 6:** Effect of augmentation scheduling on performance of proposed UDA framework.

Strategy	Augmentation	mIOU (%)					
		S→S	S→A	S→B	S→C	S→D	Average
Baseline	–	73.9	68.36	56.86	42.33	53.91	55.36
Vanilla (0.3)	Geometric (G)	74.37	82.3	70.59	55.98	72.67	70.39
	Noise/Dist. (D)	77.59	78.49	74.63	55.83	73.42	70.59
	Collage (C)	80.35	84.7	71.33	53.91	75.19	71.28
	G+D	81.03	74.9	66.14	53.55	65.67	65.06
	G+D+C	79.93	75.67	68.54	52.36	63.34	64.97
Progressive (0.3)	G+D	82.59	83.57	75.85	57.75	78.36	73.88
	G+D+C	**88.5**	**91.02**	**83.11**	**60.3**	**81.1**	**78.88**

Bold and underlined values represent best and second-best values, respectively.

However, when using all augmentations at once throughout the training process (i.e., G+D+C), the network’s performance drops as compared to when only using G+D. We believe this is because the augmentations are quite strong from the start of training, making it difficult for the network to learn important distinguishing features. To overcome this, we deployed the proposed augmentation scheduling strategy, which fully activates each augmentation after a certain number of epochs (set by the user as a hyperparameter), so that the network can easily and quickly learn simple representations at the start of training. At the end of training, when all augmentations are fully activated, these stronger regularizations make learning more difficult for the CNN and improve its robustness.

As can be seen in **Table 6**, even without using collage augmentation, the augmentation scheduling algorithm improves the average mIOU by almost 9% compared to vanilla G+D+C. When using all three types of augmentation with progressive strategy, the results improvement is almost 14% as compared to the vanilla augmentation strategy and about a 22% increase when using no augmentation at all.

### 4.5 Training and loss curves across domains

In **Figure 9**, the graphs illustrate the training loss (source domain only) and accuracy curves for the proposed domain adaptation for the source domain and average of all the target domains. The system successfully adapted from one domain to the other and was able to effectively recognize both crops and weeds across various seeding bed systems.

## 5 Visual analysis

The qualitative results of the proposed method are illustrated in **Figure 10**. The figures present some examples of the system’s qualitative performance on the testing dataset from the target and source domains. The system is capable of identifying crops and weeds effectively across different fields, even with varying densities of weeds and different seeding systems. Our approach is robust in addressing the recognition of crops (beans) and weeds, even in complex target (unseen) field environments used for domain adaptation. The underlying reason behind this performance is the utilization of deep feature alignment and augmentation scheduling algorithm which allows the system to incorporate more robust features and context information, leading to more stable and reliable segmentation results.

## 6 Conclusion

In this research, we presented an approach for unsupervised domain adaptation for crop-weed recognition in an unseen field environment. The main challenge in creating an automatic weed management system is the varying visual appearance of weeds based on factors such as lighting, weather, soil, and seeding bed type. We proposed to address this problem by minimizing the entropy of the network on target domain dataset and aligning the features of both domains through deep feature alignment. Our proposed framework, which is trained in an end-to-end fashion, consists of two main components: a segmentation network for feature extraction and robust entropy minimization and a discriminator network for adversarial training to generate target domain features as close as possible to the source domain. Additionally, we proposed the use of a augmentation scheduling strategy that starts with weak augmentations for quick adaptation to the source domain dataset and gradually increases to stronger augmentations for improved robustness and generalizability. We also demonstrated that the use of collage augmentation improves performance on target domains even further. Our extensive evaluation across four different fields with various environments and plant seeding systems showed an overall performance gain of approximately 10% mIOU on average compared to the baseline. Furthermore, using just one image for fine-tuning in a few-shot SDA setting, our network achieved almost similar performance to that of a fully supervised network, i.e., 80.53% vs 83.6%. A potential direction for future research would be to explore the adaptation of the model for recognition of multiple crops and weeds.

## Data availability statement

The original contributions presented in the study are publicly available. This data can be found here: https://github.com/Mr-TalhaIlyas/ARUFE.

## Author contributions

TI and HK did conceptualization, investigation, methodology, validation, formal analysis. TI wrote the original draft and then revised. TI and HK supervised, reviewed and edited manuscript. HK, YJ and OW secured funding. TI and JL collected and labelled data, performed investigation and visualization. All authors discussed the results and contributed to the final manuscript. All authors contributed to the article and approved the submitted version.
